# From the Farm to the Lab: How Chicken Embryos Contribute to the Field of Teratology

**DOI:** 10.3389/fgene.2021.666726

**Published:** 2021-07-22

**Authors:** Gabriela Elis Wachholz, Bruna Duarte Rengel, Neil Vargesson, Lucas Rosa Fraga

**Affiliations:** ^1^Postgraduate Program of Genetics and Molecular Biology, Department of Genetics, Universidade Federal do Rio Grande do Sul, Porto Alegre, Brazil; ^2^Laboratory of Genomic Medicine, Experimental Research Center, Hospital de Clínicas de Porto Alegre, Porto Alegre, Brazil; ^3^Teratogen Information Service, Hospital de Clínicas de Porto Alegre, Porto Alegre, Brazil; ^4^School of Medicine, Medical Sciences and Nutrition, Institute of Medical Sciences, University of Aberdeen, Aberdeen, United Kingdom; ^5^Department of Morphological Sciences, Institute of Health Sciences, Universidade Federal do Rio Grande do Sul, Porto Alegre, Brazil; ^6^Postgraduate Program in Medicine: Medical Sciences, Universidade Federal do Rio Grande do Sul, Porto Alegre, Brazil

**Keywords:** congenital malformations, preclinical trials, gene expression, teratogens, embryonic anomalies, drug/medicine safety, ZIKV, thalidomide

## Abstract

Congenital anomalies and its causes, particularly, by external factors are the aim of the field called teratology. The external factors studied by teratology are known as teratogens and can be biological or environmental factors for example, chemicals, medications, recreational drugs, environmental pollutants, physical agents (e.g., X-rays and maternal hyperthermia) and maternal metabolic conditions. Proving the teratogenicity of a factor is a difficult task requiring epidemiology studies as well as experimental teratology evidence from the use of animal models, one of which is the chicken embryo. This model in particular has the advantage of being able to follow development live and *in vivo*, with rapid development hatching around 21 days, is cheap and easy to manipulate and to observe development. All this allows the chicken embryo to be used in drug screening studies, teratogenic evaluation and studies of mechanisms of teratogenicity. The chicken embryo shares morphological, biochemical and genetic similarities with humans as well as mammalian species, making them ideal to ascertain the actions of teratogens, as well as screen drugs to test for their safety. Pre-clinical trials for new drugs are carried out in rodents and rabbits, however, chicken embryos have been used to screen new compounds or analogs of thalidomide as well as to investigate how some drugs can lead to congenital malformations. Indeed, the chicken embryo has proved valuable in understanding how many congenital anomalies, seen in humans, arise following teratogen exposure. The aim of this review is to highlight the role of the chicken embryo as an experimental model for studies in teratology, exploring its use in drug screening studies, phenotypic evaluation and studies of teratogenic mechanisms of action. Here, we discuss many known teratogens, that have been evaluated using the chicken embryo model including some medicines, such as, thalidomide, valproic acid; recreational drugs including alcohol; environmental influences, such as viruses, specifically ZIKV, which is a newly discovered human teratogen. In addition, we discuss how the chicken embryo has provided insight on the mechanisms of teratogenesis of many compounds and also how this impact on drug safety.

## Introduction

In humans, data regarding congenital anomalies (CA) induced by teratogens are from observational studies, in which case reports and epidemiological studies indicate an increase of the rate of some malformation and potential risk factors. Due to the fact that CA can cause infant and childhood death, illness and disabilities, it is important to have such methods to allow further investigation of their causes, risk factors and mechanisms [World Health Organization (WHO), 2020a]. Such investigations help directly in the development of strategies to prevent further occurrence, therapeutic interventions and deaths from CAs.

Nowadays many tools and approaches are utilized to study the causes and mechanisms that underlie a CA, from a range of cell culture assays to multiple animal and embryo models. In the context of CA, the chicken embryo model is very relevant since a lot of what is known about embryo development comes from studies with this model (Davey et al., 2018). In addition, many characteristics of the chicken embryo make it very attractive. For example, the chicken embryo offers the advantage of being able to study development live and *in vivo* throughout gestation (Davey et al., 2018). The chicken embryo also has the advantage of allowing easy administration of chemicals, compounds, external agents that may or are alleged to cause CA to determine their actions upon the embryo. This then allows the study of such CA and enables multiple analyses, genetic and morphological, to be performed in order to understand how the CA was caused and shed light on therapeutic strategies as well as how they could be prevented.

This review aims to highlight the role of the chicken embryo as an experimental model for studies in teratology. For that, we first, review congenital anomalies, teratogenesis, experimental teratogenesis and the advantages of the chicken embryo for such research. Secondly, we discuss examples of how and why the chicken embryo is used for evaluation of risks of exposure to different teratogens, understanding mechanisms of teratogens and also as a tool for preclinical trials in drug screening.

## Congenital Anomalies and Teratogenesis—A Review

Congenital Anomalies are structural, metabolic or functional abnormalities existent on birth and can be diagnosed prenatally, at birth or even later [Christianson et al., 2006; World Health Organization (WHO), 2020a]. CAs represent around 25% of infant deaths in the United States of America and 15–39% in Latin America [World Health Organization (WHO), 2020b]. Worldwide, out of 2.5 million deaths of children under 28 days of age in 2017, 283,582 were due to CA [World Health Organization (WHO), 2020b]. Most incidences of CA are from unknown causes, representing 50–75% of all cases [Brent, 2001; World Health Organization (WHO), 2020a]. The known causes are divided into chromosomal and genetic factors, environmental factors, such as exposure to a teratogen or lack of some nutrient in the diet, or a combination between them (Moore et al., 2019). Environmental factors are responsible for around 10% of the anomalies observed (Brent, 2001).

There have been many different approaches in understanding the effects of environmental exposure to the embryo over the past century. An important landmark in teratogenesis (particularly in experimental teratogenesis) was Hale’s publication in 1935. In Hale’s studies, it was reported that pregnant pigs fed with a diet deficient in vitamin A resulted in newborns with congenital malformations (Hale, 1935). These findings were next confirmed in rats by Josef Warkany in 1946, which work underpins the Principles of Teratology postulated first by Wilson (Warkany and Schrafenberger, 1946; Wilson, 1973). From this study, further work in animal models and observations in humans indicated that environmental factors, including maternal conditions, can have important effects upon the development of the embryo and adult disease (Barker and Osmond, 1986; Calkins and Devaskar, 2011). Up until the 1940s, it was thought the placenta worked as a barrier between the embryo/fetus and the outside world, protecting it from environmental agents such as drugs and pathogens. Nevertheless, reports from children whose mothers have had Rubella during pregnancy being born with cataract showed that environmental factors can cross the placenta and damage the embryo (Gregg, 1941).

Environmental factors with the potential of causing CAs in embryos and fetuses exposed during their development are named teratogens. The science that studies the CAs caused by these teratogens and how they affect the embryo by studying pathways and mechanisms that could be caused or altered by them is known as teratology (“teratos” = monster, “logos” = study; Ujházy et al., 2012). A teratogen can be any factor that is extrinsic to embryonic development, such as recreational drugs, medications, biological agents, x-rays and maternal conditions (Brent, 2001; Fraga et al., 2016; Vargesson and Fraga, 2017; Moore et al., 2019). Alcohol, cocaine and nicotine are examples of recreational drugs that are teratogenic (Cambell, 2003; Williams et al., 2015; Holbrook, 2016). Along with recreational drugs, chemicals like mercury, lead and cadmium; medicines like valproic acid, retinoic acid, phenytoin, thalidomide (Gilbert-Barness, 2010; Vargesson and Fraga, 2017); physical agents such as radiation; and maternal conditions such as obesity, pre-eclampsia and hyperthermia, can also be teratogenic (Ferm and Carpenter, 1967; Pleet et al., 1981; Léonard et al., 1983; Bellinger, 1994; Fattibene et al., 1999; Baeten et al., 2001; Gilbert-Barness, 2010; Vargesson and Fraga, 2017).

Perhaps the most famous (or infamous) teratogen is thalidomide and an example of a more recently discovered teratogen is Zika virus (ZIKV). Thalidomide cause a group of malformations in the limbs, heart, eye, ear, and internal organs (Vargesson, 2015), and led to changes in the way medicines are tested and evaluated (Vargesson, 2013). Thalidomide was originally used as a sedative and found to also be effective in treating morning sickness. Thalidomide was thought to be safe. Tragically, more than 10,000 children were born in the late 1950’s and early 1960’s with rare and severe malformations caused by the use of thalidomide during pregnancy (Kim and Scialli, 2011; Vargesson, 2019). More recently, ZIKV is a biological agent, and teratogen, that caused Congenital Zika Syndrome in babies born to mothers who were infected with ZIKV during pregnancy (Rasmussen et al., 2016; Schuler-Faccini et al., 2016). Patients with Congenital Zika Syndrome exhibit microcephaly and other CAs (de Araújo et al., 2016; Marrs et al., 2016; Schuler-Faccini et al., 2016; Del Campo et al., 2017). Children born in areas with no ZIKV have no increased levels of microcephaly.

## Experimental Teratogenesis

The classification of an environmental factor as a teratogen is not an easy task. Wilson proposed six principles of teratology to provide a guide to determining and understanding teratogenic agents (Wilson, 1973). These include susceptibility to teratogenesis depends on the genotype, depends on the developmental stage of the embryo, how the teratogen act, the nature of the teratogen and how its absorbed its maternal/placental transfer, proposed different classifications of teratogenic insult (death, malformation, growth retardation, and functional defect) (Wilson, 1973). These principles underpin the field of teratology today. More recently, Shepard (1994) further elaborated the Wilson Principles and proposing additional criteria to guide the process of proving the teratogenicity of a factor in humans, including guidance on clinical and epidemiological findings to indicate if the occurrence of a CA in a population or a cluster of individuals is linked to an environmental influence or exposure.

The history of human teratogenesis shows that it is usually an astute clinician who observes and identifies a potentially teratogenic situation. The demonstration of biological plausibility of the teratogenic potential of a determined exposure is usually determined by *in vivo* studies, performed through experimental teratology using animal models. In addition, the study of teratogenesis using experimental models is essential to understand how a specific teratogenic agent affects embryonic development *in vivo*, to help understand the agent and find treatments or prevention of congenital anomalies. Experimental teratogenesis is fundamental to evaluate and understand the mechanisms that lead to the malformations caused by exposure to the agent. Experimental models are also important and useful for drug development when animals are used in preclinical studies to evaluate reproductive and developmental toxicity and evaluation of exposure risk to a specific agent. Studies in animals also allow control of the dose and period of exposure, variables that can apply directly to a teratogenic effect (for example, Mahony et al., 2013; Beedie et al., 2016a, 2017; Cugola et al., 2016; Vargesson and Fraga, 2017).

A wide range of animal models has been used in studies of experimental teratogenesis. The most commonly utilized experimental models are embryos of mice, rats, zebrafish, chicken, rabbit, and non-human primates (for example, Janer et al., 2008; Therapontos et al., 2009; Ema et al., 2010; Ito et al., 2010; Cassina et al., 2012; Mahony et al., 2013; Beedie et al., 2015, 2016a,b; Davey et al., 2018). The choice of the appropriate experimental model depends not only on representing the phenotypes observed in humans but also considers the length of development and the accessibility of the organs and structures potentially affected. Using multiple animal models helps confirm how an alleged teratogen acts and the damage it can cause. Many other factors help in the decision of the most suitable animal model to be used such as facility of manipulation, access to embryo for observation, ethical and founding limitations, species-specificity criteria and susceptibility to the teratogen (Cassina et al., 2012).

Due to the tragedy of thalidomide, for reproductive and developmental toxicity evaluation in preclinical studies, it is now mandatory to use multiple different animal species (Vargesson, 2013). This is important because the sensitivity to some teratogenic agents varies between different species (Janer et al., 2008; Vargesson, 2015). For example, rodents are not very sensitive to thalidomide (Janer et al., 2008; Vargesson, 2015). Interestingly, rodents are also not naturally susceptible to ZIKV infection (Grant et al., 2016), but can be induced to be susceptible (Cairns et al., 2018). The chicken embryo on the other hand is susceptible to thalidomide and also to ZIKV, making it a key model for such studies (Vargesson, 2015; Goodfellow et al., 2016; Willard et al., 2017; Thawani et al., 2018, 2020; Wachholz et al., 2021).

The establishment of experimental models using different teratogens is fundamental since they are essential for the process of understanding the pathways involved and the mechanisms of teratogenesis of each teratogen. Taking into account biological teratogens, such as ZIKV, the experimental models remain indispensable during the process of development of a drug that can treat or prevent any damage caused by exposure during pregnancy.

## The Chicken Embryo

**FIGURE 1:**
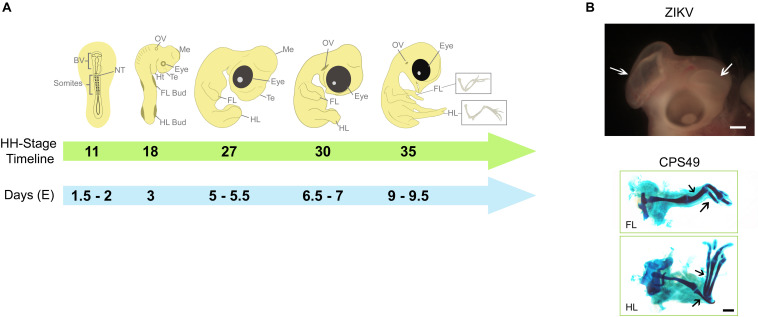
Key developmental structures of the chicken embryo and their timings. **(A)** The chicken embryo develops rapidly, the developing heart, limbs, eyes and brain can be used to evaluate teratogenic potential of teratogens such as thalidomide and Zika virus. By HH Stage 11 (Embryonic day E1.5-2), brain vesicles are developing and exposure to potential teratogens that might affect brain development may confirm their harmfulness, which become more evident by HH Stages 27 - 30. By HH Stage 18 (E3), limb buds can be observed and used to test substances with potential to affect limb development, which can be observed throughout development and confirmed by HH Stage 35 (E9-9.5), when cartilage elements can be better visualized. **(B)** Examples of teratogenic exposure and their effects on the development of chicken embryo; ZIKV induces to brain defects (white arrows; described previously in Wachholz et al., 2021) and CPS49, an antiangiogenic analog of thalidomide, which causes limb defects with reduction or absence of cartilage elements (black arrows; described previously in Therapontos et al., 2009). Images in B are from archives from Lucas Fraga’s Lab (ZIKV) and Neil Vargesson’s Lab (CPS49). BV, Brain Vesicles; NT, Neural Tube; FL, Forelimb; HL, Hindlimb; Te, telencephalon; Me, Mesencephalon; Ht, Heart; OV, Otic Vesicle.

The chicken embryo (*Gallus gallus;* Figure 1) has been used for more than two millennia as an experimental model for studies in a wide range of fields including naturalism, art, philosophy and the beginning of biological sciences trying to answer the most fundamental questions of life, for example how an embryo form (Stern, 2004). For instance, chicken embryo development was first studied in the Egyptian era around 300 BC, and by the Greek scientist, Aristoteles who performed studies focused on anatomy and embryo morphology. Even Leonardo da Vinci used chicken embryos to explore anatomy and embryo development (Stern, 2004). During the last 50 years, in particular, studies with chicken embryos have being contributing to the knowledge about some of the most important concepts in developmental biology particularly in limb development and nervous system development and more recently drug safety (Vargesson et al., 1997; Lewandoski et al., 2000; Stern, 2004; Davey and Tickle, 2007; Towers and Tickle, 2009; Vergara and Canto-Soler, 2012; Mahony and Vargesson, 2013; Beedie et al., 2016a,b; Davey et al., 2018). Indeed, the chicken embryo was fundamental to the work of the neurobiologist Rita Levi-Montalcini who won a Nobel Prize in 1986. The chicken embryo was also one of the first model organisms to study the actions of the most infamous teratogen, thalidomide (Jurand, 1966; Vargesson, 2009; Davey et al., 2018).

The importance, influence and wide use of the chicken embryo is due to the fact that they are easy to manipulate and to maintain, they are a large embryo and thus, easy to visualize live and *in ovo* and follow development throughout gestation. They are also low cost and can be obtained in large numbers. By using chicken embryos, it is possible to analyze the development macroscopically and without embryonic and/or maternal sacrifice, which makes the analyses easier and faster when compared to other models that need long processing procedures. The embryo is isolated from its mother in an egg, and develops rapidly and is fully formed by day 10 and hatches around day 21. Together this makes the chicken embryo a very favorable model organism and supports the “3Rs”—the replacement, refinement and reduction of animals in research, as there is no need to euthanize the mother to obtain embryos, unlike mammalian species (Beedie et al., 2017; Vargesson and Fraga, 2017; Davey et al., 2018).

It is important to highlight that there are differences between chicken and mammalian embryo development. Chicken embryo development is external to the body in a self-contained egg with a yolk sac, which contains nutrients and molecules with a maternal origin. Unlike mammals, including humans, where the embryo develops inside the body, with a placenta supplying nutrients, and as there is also a maternal liver, means drugs can also be metabolized before arriving at the embryo. The chicken embryo not having a maternal metabolism allows scientists to understand the effect of some compounds in the embryo with the absence of maternal influence as well as help determine if a drug’s action requires maternal metabolism. Phenytoin, for instance, is an anti-epileptic drug that exerts its teratogenic effects through its arenes oxides metabolites (Spielberg et al., 1981; Strickler et al., 1985). Chicken embryos present with neural tube defects when they are exposed to phenytoin (Temiz et al., 2009). This suggests that the effect of phenytoin is not related exclusively to maternal metabolism and, therefore, reinforces the usefulness of the chicken embryo model. In addition, because of species-specificity of teratogenesis, some agents are not teratogenic in rodents, such as thalidomide (Vargesson, 2019). This is why chickens (and other species) can and have helped work out the actions of these drugs and confirm their safety (Vargesson, 2015). Therefore, as a method of understanding how a drug acts and determining its potential to cause teratogenesis, the chicken embryo remains powerful.

Chicken embryo developmental stages were extensively described by Hamburger and Hamilton (1951), and which are used today when studying chicken embryo development. Their description and delineation of a system of stages also help to compare chicken embryo development with other animal species and also human development, allowing research in human congenital anomalies to be performed in this model. For example, following the first 3 days of development when gastrulation and neurulation occurs, heart development quickly follows (Hamburger and Hamilton, 1951). Followed rapidly from 3 days, by the limb buds, together with the formation of other organs like eyes, ear and brain (Hamburger and Hamilton, 1951; Davey et al., 2018; Vilches-Moure, 2019; Figure 1). Together with the comparable embryology and physiology, there is a high genome conservation between mammals and chicken. Furthermore, the chicken genome is sequenced (Wallis et al., 2004).

One of the limitations of the use of chicken embryos is its relative lack of genetic tractability, compared to rodents. This is due to fertilization and early developmental events occurring inside the hen, followed by the egg shell being produced before being laid. This makes techniques like targeted gene knockouts through homologous recombination very difficult. However, it is possible to misexpress genes using the RCAS virus system (Logan and Tabin, 1998) and inhibit genes using RNAi (Krull, 2004). Also, more recently genomic editing techniques using CRISPR/Cas9 have started to be used chicken embryos, allowing targeted gene knockdowns (Williams et al., 2018; Chojnacka-Puchta and Sawicka, 2020). Taken all together, these techniques reinforce the usefulness of the chicken embryo in development, how drugs affect development and also genomic and molecular analyses (Burt et al., 1999; Stern, 2018).

## The Chicken Embryo in the Evaluation and Understanding of Teratogens and Drug Safety

The development of a new drug requires different steps and an important one is the prediction of the potential teratogenic effects during development (Beedie et al., 2017). In preclinical trials, drugs in development are tested in pregnant animals, more frequently in mammals, which have a more similar physiology to humans. Since the thalidomide tragedy and the discovery of the species-specificity property of teratogenesis, it is necessary to perform drug screening in more than one species, including a rodent and a non-rodent, to attest its safety during development (Vargesson, 2013). Presently, testing of new drugs is mainly carried out in rodents and rabbits. However, chicken embryos have proved useful to understand actions of drugs linked to causing developmental problems and understanding a suspected teratogens mechanism of action, for example, thalidomide (Jurand, 1966; Knobloch et al., 2007; Therapontos et al., 2009; Vargesson, 2015; Beedie et al., 2016a,b). In addition, the advantage of live and *in vivo* observations of the chicken embryo following drug exposure, allows scientists to understand the drugs actions when applied at different timepoints and concentrations and drug actions can be followed throughout development. Chicken embryos are also being used as a preclinical screen to ascertain potential actions/side-effects of new drugs as well as screen drugs, for example thalidomide, phenytoin, ethanol, to understand their actions (Beedie et al., 2015, 2016a,b; Davey et al., 2018). This can (i) help reduce the number of rodents required in preliminary drug screening, as studies in the chicken embryo may indicate harmful effects and thus, not to be followed up in rodents; and (ii) give some insight into potential mechanisms of action of the drug as well as safety.

The chicken embryo enables analysis of the effects of a drug throughout development (and live and *in ovo*) including through early stages of organogenesis (Beedie et al., 2017), which is difficult in models such as rodents. Effects of drugs/compounds/agents can also be followed throughout development. As studies of the phenotypic spectrum and teratogenic risk of an agent in humans are limited to epidemiological analyses, this can be carried out in the chicken embryo as can a dose-response analysis and observation of the effects of varying the timing of exposure upon development, to determine the most sensitive time of development for each agent. This further underlines the importance of the chicken embryo as an excellent experimental model to follow and determine the risks of a teratogen (Wachholz et al., 2021).

For example, the chicken embryo model can be used to mimic human phenotypes after teratogen exposure, as it is the recent case of Zika virus. Wachholz et al. (2021) was able to mimic the microcephaly phenotype observed in humans after Zika virus infection during development in chicken embryos using a non-invasive method of infection. Chicken embryos have been used as a model for ZIKV infections since 1951 (Taylor, 1952) and today there are multiple research groups using the chicken embryo as a model of Congenital Zika Syndrome (Goodfellow et al., 2016; Willard et al., 2017; Thawani et al., 2018, 2020; Wachholz et al., 2021).

Some of the drugs/medicines that have been studied in chicken embryos to identify potential and/or understand teratogenic actions include thalidomide and thalidomide analogs including pomalidomide and tetrafluorinated thalidomide analogs with anti-cancer properties (Kemper and Berger, 1962; Kemper, 1963; Jurand, 1966; Tamilarasan et al., 2006; Knobloch et al., 2007; Therapontos et al., 2009; Vargesson, 2009; Ito et al., 2010; Mahony et al., 2013; Vargesson, 2019; Beedie et al., 2015, 2016a); the new-generation anti-epileptic medication Levetiracetam (Tureci et al., 2011); the anti-epileptic medicine sodium valproate (Whitsel et al., 2002); as well as anti-angiogenic anti-cancer compounds (Beedie et al., 2015, 2016a,b; Table 1). The chicken Chorioallantoic Membrane (CAM) has been also used to screen the anti-tumorigenic activity of overexpressed proteins (Valiulytë et al., 2019) and plays an important role in other cancer and cardiovascular studies, including bioengineering (Warnock et al., 2013; Kain et al., 2014; Nowak-Sliwinska et al., 2014).

**TABLE 1:** Morphological phenotypes observed after exposure of chicken embryos to teratogens.

Teratogen	Phenotypes	References
Alcohol	Reduced body size; brain malformations; heart anomalies; ventricular septal defect; otic vesicle anomalies; Defects in the caudal region; spina bifida	Sandor and Elias, 1968; Sandor, 1968; Fang et al., 1987
Cadmium	Limb, heart, vasculature, liver, neural tube, somites, and reproductive system anomalies	Cullinane et al., 2009; Yamamoto et al., 2012
Retinoic acid	Heart anomalies; cardia bifida; facial anomalies; limb anomalies.	Tamarin et al., 1984; Osmond et al., 1991
Thalidomide and thalidomide analogs	Limb and digit anomalies; amelia; microphthalmia; reduced body size; hemorrhaging	Stephens, 2009; Therapontos et al., 2009; Ito et al., 2010; Beedie et al., 2016a
Valproic acid	Neural tube defects; Cardiovascular anomalies; Craniofacial defects; limb and skeletal malformations	Whitsel et al., 2002; Hsieh et al., 2012, 2014
Zika virus	Reduced brain size; reduced body size; mesencephalon, telencephalon and eye reduction size; mesencephalon malformations; understaged embryos; inner ear dysmorphogenesis	Goodfellow et al., 2016; Thawani et al., 2018, 2020; Willard et al., 2017; Wachholz et al., 2021

Other examples of teratogens studied in chickens include studies looking at valproic acid exposure which can cause neural tube defects in the chicken embryo, together with cardiovascular anomalies (Whitsel et al., 2002; Hsieh et al., 2012, 2014). Limb and skeletal anomalies are also observed after exposure of valproic acid and retinoic acid (Tamarin et al., 1984; Whitsel et al., 2002). Congenital anomalies of the heart and cardiovascular system were also described after chicken embryo exposure to alcohol and retinoic acid (Fang et al., 1987; Whitsel et al., 2002). The exposure of thalidomide in chicken embryo has shown to cause limb anomalies, resembling the ones observed in humans, such as amelia (Stephens, 2009; Ito et al., 2010; Beedie et al., 2016a), phocomelia and radial dysplasia (Therapontos et al., 2009; Davey et al., 2018).

The chicken embryo has also been used to study treatments to prevent congenital anomalies caused by exposure of teratogens. For example, (i) damage caused by valproic acid, can be prevented or can be rescued by exposure with resveratrol, vitamin E, folic acid, n-acetylcysteine and vitamin C (Hsieh et al., 2012, 2014); (ii) damage caused by thalidomide has been prevented or rescued through application of nitric oxide (Siamwala et al., 2012); (iii) Cadmium induced damage can also be rescued by nitric oxide exposure (Veeriah et al., 2015).

Furthermore, the chicken embryo has also been used to study the consequences of teratogens in childhood and adult life (i.e., late-onset effects). For example, studies have used this model to analyze behavioral and memory impairments after prenatal exposure of factors, including ethanol, morphine, hypoxia, malnutrition, D-cycloserine, and others (Dose et al., 1995; Steele et al., 1996; Rodricks et al., 2004; Che et al., 2005; Rao and Chaudhuri, 2007; Smith et al., 2011; Mohammad et al., 2012). It has been observed that ethanol and morphine can affect long term memory in chicken when treated prenatally (Che et al., 2005; Rao and Chaudhuri, 2007). Along with memory, behavioral effects are also observed (Dose et al., 1995; Smith et al., 2011; Mohammad et al., 2012; Johnsson et al., 2016). Some of the outcomes observed included increase of fearfulness and tonic immobility and reduction of reflexive motor activity. Interestingly, the chicken was already used as a genomic model for tonic immobility and anxiety behavior (Johnsson et al., 2016; Fogelholm et al., 2019). Whether such late-onset effects are caused by brain malformations or if they occur in an independent manner, this remains to be further clarified. Nevertheless, chicken embryos are useful in investigating these outcomes.

Although the chicken embryo is a good model for understanding development and for understanding drug action it is not a standard system for pre-clinical drug screening. Consequently, most of the drugs studied in the chicken embryo the pharmacokinetic information is unclear. However, while the chicken embryo is not a standard tool for pharmacokinetics, it has been used in studies of toxicogenomics related to environmental exposure of bisphenol A alternatives, organic flame retardants and 6-OH-BDE47, which is a Hydroxylated polybrominated diphenyl ether (Peng et al., 2016; Pagé-Larivière et al., 2018; Sharin et al., 2021), indicating this is an area of expansion in the chicken embryo field, and which would further enhance its use in understanding pharmacokinetics.

## The Chicken Embryo and Understanding Mechanisms of Teratogenesis

While many teratogens have been identified that are known to cause birth defects, how they cause the birth defects has remained unclear. Given the advantages of the chicken embryo, many teratogenic agents mechanistic bases have begun to be identified and include thalidomide, valproic acid, alcohol, cocaine, nicotine, retinoic acid and cadmium just to mention a few (Wiens et al., 1992; Venturini and Sparber, 2001; Cullinane et al., 2009; Therapontos et al., 2009; Ito et al., 2010; Hsieh et al., 2012; Yamamoto et al., 2012; El-Beltagy et al., 2015). Understanding the mechanisms of teratogenicity of a factor can shed light on the biological action of the compound as well as how it causes congenital anomalies, which can lead to potential ways of making safer drugs. In addition, the more we understand how congenital anomalies occur strategies of prevention can be determined.

Using chicken embryos, it was shown that one of the effects of alcohol on embryo development is due to the activation of a G-protein and a calcium-dependent mechanism of apoptosis (Table 2). This process was observed to cause effects in the brain and neural tube (Garic-Stankovic et al., 2005; Flentke et al., 2014). Chicken embryo exposure with alcohol was further used to understand the malformations observed in Fetal Alcohol Syndrome (FAS), for example, the craniofacial anomalies (Smith, 2008). Therefore, the chicken embryo is a good model to understand how organs and tissues are affected and which molecular pathways are disrupted (Garic-Stankovic et al., 2005; Smith, 2008; Flentke et al., 2014).

**TABLE 2:** Examples of mechanisms of teratogenesis of some teratogens using chicken embryos as experimental models.

Teratogen	Mechanism	References
Alcohol	Activation of G-protein; Apoptosis induction; CaMKII activation; Dysregulation of beta-catenin signaling; Reduction in neural cells proliferation.	Garic-Stankovic et al., 2005; Giles et al., 2008; Flentke et al., 2014
Thalidomide	Antiangiogenesis; Induction of oxidative stress; Thalidomide binding to Cereblon; Upregulation of bmp and dkk1; Downregulation of fgf10 and fgf8; Induction of apoptosis.	Knobloch et al., 2007; Therapontos et al., 2009; Ito et al., 2010
Valproic acid	Inhibition of angiogenesis; Induction of oxidative stress; Inhibition of hdac; Reduction of pax-2 and pax-6 levels; Downregulation of sod and rbp4.	Whitsel et al., 2002; Chuang et al., 2012; Hsieh et al., 2012, 2013
ZIKV	Reduced transcript level of shh, bmp7 and fgf8; Patched1 reduction.	Thawani et al., 2018

*CaMKII, Ca^2+^/calmodulin-dependent protein kinase II; bmp, bone morphogenetic protein; dkk1, Dickkopf WNT Signaling Pathway Inhibitor 1; fgf10, fibroblast growth factor 10; fgf8, fibroblast growth factor 8; hdac, histone deacetylase; sod, superoxide dismutase; rpb4, retinol binding protein 4; fgf8, fibroblast growth factor 8; bmp7, bone morphogenetic protein 7; shh, sonic hedgehog.*

The chicken embryo is also known as one of the models that has helped to understand how thalidomide caused such severe damage to the human embryo. While the precise mechanisms remain to be fully determined, of the many proposed mechanisms, there are three which are considered more likely to be the cause of thalidomide’s teratogenicity (D’Amato et al., 1994; Knobloch et al., 2007, 2017; Therapontos et al., 2009; Ito et al., 2010; Vargesson, 2013, 2015, 2019; Vargesson and Hootnick, 2017). They include anti-angiogenesis, oxidative stress induction and the binding of thalidomide to a protein named Cereblon. The chicken embryo demonstrated the antiangiogenic potential of thalidomide as a cause for limb deformities (Therapontos et al., 2009; Vargesson, 2009; Davey et al., 2018). Furthermore, the oxidative stress and cell death induction mechanism was tested in chicken embryos, where following thalidomide exposure upregulation of *bone morphogenetic protein* (*bmp*) and *Dickkopf WNT Signaling Pathway Inhibitor 1* (*dkk1*) genes, involved in cell death was observed (Knobloch et al., 2007). Other studies in chicken embryos have also observed the downregulation of *fgf10* and *fgf8* mRNA, and induction of apoptosis (Knobloch et al., 2007; Therapontos et al., 2009; Ito et al., 2010). More recently the binding of thalidomide to Cereblon protein was first described using the chicken embryo as one of the approaches (Ito et al., 2010). Cereblon is a ubiquitin ligase whose function is to tag and remove unwanted molecules. The binding of thalidomide with Cereblon results in a disruption of cellular signaling. Precisely how the thalidomide/Cereblon complex causes the actual tissue damage is unclear, and there is also evidence that thalidomide may too be acting in a cereblon-independent mechanism, particularly with respect to thalidomide’s actions on the blood vessels (Beedie et al., 2020; Peach et al., 2020).

The chicken embryo has also shed light on how Valproic acid, used in humans to treat epilepsy, can cause embryonic malformations. In chicken embryos exposed to Valproic acid, damage to the embryo is rapidly observed (notably to limbs and eyes) through inhibition of angiogenesis, induction of oxidative stress and inhibition of histone deacetylase and key genes involved in tissue development including Pax2 and Pax6 (Whitsel et al., 2002; Chuang et al., 2012; Hsieh et al., 2012; Table 2).

Finally, although the molecular mechanisms of ZIKV teratogenesis are still to be fully determined, some important advances have been made using chicken embryos to understand how exposure to ZIKV during development causes malformations and the molecular basis of this. The first experiments aiming to understand the teratogenesis of ZIKV using as experimental models chicken embryos showed microcephaly, reduction on the head size, indicating disrupted brain growth, reassembling the CZS patients phenotype and giving a clue of which molecular mechanism could be involved (Thawani et al., 2018). Thawani et al. (2018) have demonstrated that ZIKV impairs the expression of important signaling molecules such as *shh*, *bmp7*, and *fgf8* in specific regions of the developing brain. Such findings are important to increase knowledge about this recently discovered human teratogen, however, further studies are necessary to better define the mechanisms of teratogenesis of this virus.

## Conclusion and Perspectives

The chicken embryo model allows live and *in ovo* studies and analyses throughout development to define and evaluate potential risks of compounds, determine teratogenic phenotypes and understand and describe the mechanism involved in its teratogenic process. All this is essential to create and design strategies, as well as preventive and therapeutic measures in order to avoid the occurrence of congenital anomalies. And equally important to help design safer drugs and medicines in the future.

Thalidomide is a great example of the importance of the chicken embryo, since it helped to understand the mechanisms of teratogenesis and to the identification and screening of safer alternative drugs (Jurand, 1966; Beedie et al., 2016a; Vargesson, 2019). ZIKV is also another good and recent example of this experimental model, since the chicken embryo is helping to understand the mechanisms of viral teratogenesis and to aid design prevention strategies to avoid the occurrence of malformations through the screening of new drugs aimed at ameliorating the ZIKV actions on the embryo.

Finally, the knowledge obtained through chicken embryos permits the discovery and testing of new drugs capable of avoiding or to prevent phenotypes caused by exposure to teratogenic agents during development.

## Author Contributions

All authors listed have made a substantial, direct and intellectual contribution to the work, and approved it for publication.

## Conflict of Interest

The authors declare that the research was conducted in the absence of any commercial or financial relationships that could be construed as a potential conflict of interest.
